# Exosomes/Extracellular Vesicles and Targeted Tumor Immunotherapy

**DOI:** 10.3390/ijms25105458

**Published:** 2024-05-17

**Authors:** Chiara Corrado, Simona Fontana

**Affiliations:** Department of Biomedicine, Neurosciences and Advanced Diagnostics, University of Palermo, 90133 Palermo, Italy; simona.fontana@unipa.it

This Special Issue intends to underscore several topics related to cellular signaling transduction, considering the consensus that nowadays, the best scientific approach for the prevention, diagnosis, and treatment of several diseases is the study of the regulatory networks that determine the response to therapy and the maintenance of homeostasis or its dysregulation.

The study of cellular signaling pathways allows us to understand the subtle balance between healthy and diseased states, and how the deregulation of a specific cell-signaling pathway may lead to a specific disease. This is of fundamental importance for the generation of personalized therapies that target dysregulated mechanisms.

Despite the existence of many studies, the scientific community is now focusing on the comprehension and deeper analysis of genetic susceptibility to diseases, such as metabolic syndromes, cancers, cardiovascular, and neurodegenerative diseases.

Fernanda Pereira Fernandes describes the correlation between polymorphisms of inflammasome genes and several infectious and non-infectious diseases to clarify the main role of the inflammasome-signaling pathway in the progression of pathologies such as HPV or HIV, metabolic syndromes, cancers, and autoimmune diseases [1].

Pisetsky analyzes the pathogenesis of autoimmune diseases (ADs), focusing on the strict correlation between genetic predisposition and autoimmunity, and discusses how autoantibody expression may help practitioners engage in early diagnosis and accelerate the initiation of personalized therapy [2]. These two examples clearly elucidate that, to date, greater interest has been directed to autoimmune diseases (ADs) and to the dissection of signaling pathways that regulate them, finally focusing on personalized therapy. Nowadays, ADs represent a public health problem that affects the quality of life of many patients worldwide [3,4,5].

Cells communicate with each other through different signaling pathways mediated by cytokines, neurotransmitters, and hormones. Moreover, a new and very efficient endocrine mechanism of communication is represented by extracellular vesicle (EV) release.

EVs are physiologically secreted by any cell type [6] and are considered important intercellular communicators that carry several different, cargos such as lipids, nucleic acids, and proteins nearby or distant to parental cells, finally affecting the functions of the receiving cells. For their specific composition, which is strictly related to the originating cells, and for their increased secretion in pathological conditions, EVs are considered excellent biomarkers for the diagnosis of several diseases, from ADs to cancer [7,8,9]. Moreover, EVs are considered to comprise an innovative delivery vehicle for natural compounds, biological components, and drugs owing to their properties and features, including their low immunogenicity, high bioavailability and stability, and capability to be targetable [10].

In as short a span as one year, more than 880 papers on targeted therapy and exosomes were published on PubMed, and these were especially focused on tumor-targeted therapy and tumor immunotherapy. Recently, Solomon et al. reviewed the role of EVs as mediators of cellular communication in oral cancers, indicating their possible application in therapy as excellent delivery vectors for developing a system of targeted therapy [8].

Li and collaborators describe an engineering strategy to improve tumor immunotherapy, presenting an interesting example of a combinatory strategy comprising EVs and nanomaterials to induce or enhance the effects of a potential therapeutic system. Mouse H22 hepatocellular carcinoma cells were used as a bioreactor to produce Tellurium nanoparticle-capsulated EVs with high HSP70 expression for photothermal-triggered tumor immunotherapy (Te@EVsHSP70). The heat energy caused by near-infrared irradiation (NIR) determines the instability of EVs and the overexpression of HSP70 on their surface. This overexpression increases tumor antigen presentation by dendritic cells (DCs), thus inducing their maturation and the infiltration of T cells into the tumor [11].

EVs are also used as vehicles of surface monoclonal antibodies that can strongly activate an antitumor immune response. With this aim, Fan and collaborators have generated a CAR T cell therapy-mimicking system through tumor dendritic cell-derived EVs engineered toward the surface expression of anti-CD3 and anti-EGFR. This system promoted efficient endogenous T cell activation and the interaction of T cells with cancer cells, enhancing solid tumor therapy [12]. Similarly, Shi and collaborators developed genetically modified EVs expressing both anti-CD3 and anti-HER2 on their surface. These synthetic multivalent antibody-targeted platforms dually target T cell CD3+ and breast cancer HER2+ cells, showing highly potent and specific anti-tumor activity both in vivo and in vitro [13]. Jang et al. generated tumor-derived re-assembled exosomes (R-Exos) for pancreatic cancer therapy. The loading of the chlorin e6 photosensitizer allows exosomes to be visualized through photoacoustic imaging; these R-Exos can efficiently generate reactive oxygen species inside tumor cells under laser irradiation and enhance cytokine release from immune cells, thus suggesting their possible role as immunotherapeutic agents [14].

In conclusion, the studies carried out using EVs modified in terms of their surface decoration and in the internal cargo clearly highlight the potential of this emerging field for improving targeted tumor immunotherapies. The strategies employed for developing engineered EVs activating molecular mechanisms that reshape the tumor immune microenvironment are complex, and optimizing these to guarantee their successful clinical translation is still a challenge.

## References

[1] Fernandes F.P., Leal V.N., Souza de Lima D., Reis E.C., Pontillo A. (2020). Inflammasome genetics and complex diseases: A comprehensive review. Eur. J. Hum. Genet..

[2] Pisetsky D.S. (2023). Pathogenesis of autoimmune disease. Nat. Rev. Nephrol..

[3] Hsieh P.H., Wu O., Geue C., McIntosh E., McInnes I.B., Siebert S. (2020). Economic burden of rheumatoid arthritis: A systematic review of literature in biologic era. Ann. Rheum. Dis..

[4] Fatoye F., Gebrye T., Svenson L.W. (2021). Direct health system costs for systemic lupus erythematosus patients in Alberta, Canada. PLoS ONE.

[5] Papadimitropoulos E., Brnabic A., Vorstenbosch E., Leonardi F., Moyano S., Gomez D. (2022). The burden of illness of rheumatoid arthritis in Latin America: A systematic literature review. Int. J. Rheum. Dis..

[6] Ibrahim S.A., Khan Y.S. (2023). Histology, Extracellular Vesicles.

[7] Fang Y., Ni J., Wang Y.-S., Zhao Y., Jiang L.-Q., Chen C., Zhang R.-D., Fang X., Wang P., Pan H.-F. (2023). Exosomes as biomarkers and therapeutic delivery for autoimmune diseases: Opportunities and challenges. Autoimmun. Rev..

[8] Solomon M.C., Chandrashekar C., Kulkarni S., Shetty N., Pandey A. (2023). Exosomes: Mediators of cellular communication in potentially malignant oral lesions and head and neck cancers. F1000Research.

[9] Chan B.D., Wong W.Y., Lee M.M.L., Cho W.C.S., Yee B.K., Kwan Y.W., Tai W.C.S. (2019). Exosomes in Inflammation and Inflammatory Disease. Proteomics.

[10] Raguraman R., Bhavsar D., Kim D., Ren X., Sikavitsas V., Munshi A., Ramesh R. (2023). Tumor-targeted exosomes for delivery of anticancer drugs. Cancer Lett..

[11] Li X., He S., Luo B., Li P., Chen X., Wu M., Song C., Liu C., Yang T., Zhang X. (2023). Engineered Extracellular Vesicles to Enhance Antigen Presentation for Boosting Light-Driven Tumor Immunotherapy. Small.

[12] Fan M., Liu H., Yan H., Che R., Jin Y., Yang X., Zhou X., Yang H., Ge K., Liang X.-J. (2022). A CAR T-inspiring platform based on antibody-engineered exosomes from antigen-feeding dendritic cells for precise solid tumor therapy. Biomaterials.

[13] Shi X., Cheng Q., Hou T., Han M., Smbatyan G., Lang J.E., Epstein A.L., Lenz H.-J., Zhang Y. (2020). Genetically Engineered Cell-Derived Nanoparticles for Targeted Breast Cancer Immunotherapy. Mol. Ther..

[14] Jang Y., Kim H., Yoon S., Lee H., Hwang J., Jung J., Chang J.H., Choi J., Kim H. (2021). Exosome-based photoacoustic imaging guided photodynamic and immunotherapy for the treatment of pancreatic cancer. J. Control. Release.

